# Six years of high-resolution monitoring data of 40 borehole heat exchangers

**DOI:** 10.1038/s41597-024-04241-9

**Published:** 2024-12-17

**Authors:** Elisa Heim, Phillip Stoffel, Dirk Müller, Norbert Klitzsch

**Affiliations:** 1 RWTH Aachen University, Computational Geoscience, Geothermics, and Reservoir Geophysics, Aachen, 52074 Germany; 2 E.ON Energy Research Centre, RWTH Aachen University, Institute for Energy Efficient Buildings and Indoor Climate, Aachen, 52074 Germany

**Keywords:** Geothermal energy, Environmental sciences

## Abstract

Ground-source heat pumps (GSHPs) coupled with borehole heat exchangers (BHEs) are energy-efficient technologies for heating and cooling buildings. However, these systems often fail to operate at their full potential due to discrepancies between the assumptions made during the design phase and the actual conditions during operation. To enhance overall GSHP performance, it is crucial to collect and analyze long-term monitoring data from operating BHE fields. To our knowledge, no long-term, high-resolution dataset of double U-tube BHEs is currently publicly available. Additionally, most studies typically monitor only the inlet and outlet of the entire ground heat exchanger rather than individual BHEs, hindering detailed performance analysis. With this data descriptor, we present a 6-year dataset from a BHE field comprising 40 BHEs, each with sensors for volume flow and inlet/outlet temperatures, recorded every 30 seconds. We believe this dataset will enhance understanding of individual BHE performance, provide validation for BHE models, and thus support better GSHP design and operation.

## Background & Summary

Ground-source heat pumps (GSHPs) use the ground as a source of energy to heat and cool indoor spaces. Compared to fossil-fuel-based heating systems, GSHPs typically offer higher energy performance and lower primary energy consumption, leading to reduced emissions^1,2^. Borehole heat exchangers (BHEs) are one of the most common and versatile systems for exchanging heat with the ground^3^. A BHE consists of a borehole, commonly 40 to 300 m deep, into which U-shaped pipes are inserted and backfilled with grout to create thermal contact with the ground. During operation, water or a glycol mixture circulates through the pipes to supply the source side of a heat pump or to enable free cooling without the use of a heat pump. Because the average ground temperature along the BHE remains relatively constant throughout the year, ground-source heat pumps operate with high efficiency for both heating and cooling buildings.

To equip a building with a ground-source heat pump using BHEs, it is necessary to determine the optimal number and length of BHEs required to meet the building’s energy demand. During this so-called design stage, the heat exchange of a BHE configuration is simulated over 20–30 years of operation^4^, and the fluid temperatures in the final year are evaluated to ensure they remain within region-specific temperature thresholds. This design simulation requires information about the building’s energy demand and the ground’s thermal properties, both of which are challenging to predict accurately over such an extended time period. Changes in heating or cooling demand lead to a different thermal exchange in the ground, and subsurface heat exchange is inherently uncertain^5^. Effective thermal properties—especially those influenced by groundwater flow—are difficult to predict^6^, as they can fluctuate significantly over time due to changes in environmental conditions and groundwater level fluctuations. Additionally, simulation models used for design often assume a quasi steady-state thermal exchange process between fluid and ground^7^, while the real operation is often characterized by shorter, cyclic operation phases. A recent study demonstrated that neglecting these short-term effects in the design stage leads to an oversized BHE field^8^, thus influencing investment and operation cost^9^. All in all, the numerous uncertainties and assumptions result in a mismatch between the thermal performance calculated during the design stage and the actual performance during operation.

To quantify uncertainties in BHE modeling and understand real operation strategies, analyzing data from operating BHE fields is crucial. Monitoring data can reveal whether the building load profile assumed during design accurately reflects the actual load profile during operation. If discrepancies are identified, modifications can be made to prevent ground thermal imbalance^10^. Additionally, monitoring data enables the validation and comparison of borehole heat exchanger models^11^ and supports the optimization of operational strategies to reduce energy costs^12^. It also helps in identifying malfunctions within the system control^13^. Therefore, monitoring data is essential for gaining insights into operating conditions and for developing methodologies that enhance BHE design and operational efficiency.

However, easily accessible, detailed monitoring data of BHEs are sparse. Table 1 provides an overview of studies analyzing or providing BHE monitoring data. Of 19 reviewed studies, only three actually provide open-access data. For seven studies, the data is available upon request, while the remaining nine studies do not include a data availability statement. Besides availability, most studies only record the aggregated inlet and outlet temperatures of the entire ground heat exchanger (comprising several BHEs), which is insufficient for refining BHE models and optimizing operation. Moreover, data over extended timespans (exceeding three years) is limited, and the coarse time resolution (one hour) constrains the validation of optimal control problems that require high-resolution, long-term data. In addition, information about sensors, their exact placement, and their uncertainties is not always complete.

**Table 1:** Review of studies gathering or analyzing BHE monitoring data and data availability.

Monitored system	Sensors in GHE (temperature and flow rate)	Data period and resolution	Data available?
University building in Leicester (UK) with 56 single-U tube BHEs, 100 m deep^13,29^	Four PT100 fluid-T sensors (four-wired, calibrated), one clamp-on ultrasonic flowmeter (calibrated, accuracy 0.5%)	Initial three years from Jan. 2010, 1 min interval	yes^30^
Commercial building in Urbino (IT) with six single-U tube BHEs, 100 m deep^31^	Twelve PT100 fluid-T sensors (sensitivity 0.05 °C), one thermophreatimeter (sensitivity 0.1 °C), ten PT100 ground-T sensors	Four years from Oct. 2010, 2 h interval	N/A
Municipality Hall of Pylaia (GR) with 21 single-U tube BHEs, 80 m deep^32,33^	PT100 film-type 4-wire fluid-T sensors, ultrasonic flow meter	Initial eight years from Jan. 2003, 10 min interval	N/A
Archive in Shanghai (CN) with 280 single-U tube BHEs, 80 m deep^34^	Fluid: temperature automatic recorders (accuracy ±0.5 °C), ultrasonic flowmeter (accuracy 1–5%)	Two years from Dec. 2008, 1 min interval	N/A
University building in Valencia (ES) with 6 single-U tube BHEs, 50 m deep^35–37^	14 PT100 fluid-T sensors (four-wired, accuracy ±0.1 °C), 21 PT100 ground-T sensors, 1 Coriolis mass flow meter (accuracy ±0.1%)	initial eleven years from Feb. 2005, 1 min interval	yes^38^
Student apartments in Stockholm (SE) with 11 single-U tube BHEs, 225–350 m deep, 1 coaxial research BHE, 100 m deep^39^	Fluid: Produal TEAT LL-N, accuracy ±0.3 °C, ground: fiber optic cables, ultrasonic flowmeter	Half a year from May 2019, 1 h interval	upon request
Office building in Stockholm (SE) with 130 single-U tube BHEs, 230 m deep^40^	Fluid: thermistors (4 wires), flow: magnetic induction (accuracy ±0.5%)	Three years from Jan. 2017, 1–5 min interval	upon request
Club house in Gothenburg (SE) with 1 single-U tube BHEs, 230 m deep^41^	Fluid: Ten PT100 (calibrated, accuracy <0.5 °C), flow: 6 vortex (accuracy ±3%)	Three years from Jan. 2014, 2 min interval	upon request
Student center in Stockholm (SE) with 20 single-U tube BHEs, 200 m deep^19,42^	Two fluid temperature sensors (accuracy <0.5 °C), flowmeter (accuracy ±5%)	Five years from Jan. 2016, 1 h interval	yes^43^
Office building (DE) with 25 double-U tube BHEs, 100 m deep^44^	Fluid and ground: PT100/ PT500 (accuracy ΔT >0.5 °C), ultrasonic flowmeter	Nine years from Jan. 2011, 15 min. interval	upon request
Office building in Nürnberg (DE) with 18 double-U tube BHEs, 80 m deep^45^	Fluid: PT100 (accuracy ±0.05 °C), ground: TLC Meter (accuracy ±2%), flow: Promag 53 P (accuracy ±0.2%)	Four years, 10 min interval	N/A
University building in Timisoara (RO) with 1 single-U tube BHE, 80 m deep^46^	Fluid: 2 PT500 (accuracy ±0.15 °C), ultrasonic flowmeter	Two years from Jan. 2012	N/A
School building in Busan City (KR) with 24 single-U tube BHEs, 175 m deep^47^	Fluid: T-type thermocouples	Half a year from Mar. 2007	N/A
Residential building in Lugano (CH) with 13 U tube BHEs, 200 m deep^48^	Ground: 15 T-type thermocouples (calibrated, accuracy <0.3 °C), five electromagnetic flowmeters (calibrated, accuracy 2.4%)	Three years from Jun. 2016	N/A
Office building at Ponte Arche (IT) with 5 double-U tube BHEs, 125 m deep^49^	Ground: PT100, 4-wired (accuracy ±0.06 °C)	3 years from Apr. 2015, 10 min. frequency	N/A
Office building in Gelsenkirchen (DE) with 36 double-U tube BHEs, 150 m deep^50^	Fluid: PT100 (accuracy <0.5%), ultrasonic flowmeter	14 years from Jan. 2006, 15 min. interval	upon request
Hospital in Kalnes (NO) with 100 single-U tube BHEs, 250 m deep^51^	Fluid: Pt100/Pt500 (accuracy ±0.15%), flow: Metrima SVM F4/Krohne UFM3030	Five years from Jan. 2016	upon request
Office and residential building in Chongqing (CN) with 90 single-U tube BHEs, 100 m deep^52^	Fluid: T-type thermocouples (±0.5 °C accuracy), ground: RTDs (accuracy ±0.1 °C), flow: ultrasonic (accuracy 5%)	46 days from Jul. 15th and 42 days from Dec. 4th	N/A
Office building in Atlanta (US) with 12 single-U tube BHEs, 122 m deep^53,54^	Fluid: immersion (accuracy: ±0.2 °C), dual turbine flowmeter (accuracy ±2%)	Two years from Jul. 2011, 15 min interval	upon request

N/A indicates that no data availability statement was found.

With this data descriptor, we present a dataset covering six years of high-resolution monitoring data of an operating BHE field located in Aachen, Germany. The BHE field is composed of 40 individual 100 m deep double-U tube BHEs, where each BHE is equipped with its own sensor for inlet and outlet temperature and volume flow rate. Raw data is provided at 30-second intervals, along with a processed dataset that has been resampled to five-minute intervals. Parts of the data have been used to validate different modeling approaches or for theoretical studies (Table 2), but the raw data has not yet been published anywhere in a coherent and accurately documented form. The detailed, high-resolution monitoring data will serve as a foundation for future studies aimed at improving BHE design and identifying potentials for operation optimization.

**Table 2:** Overview of studies that worked with parts of the data presented here.

Study purpose	Data period and BHEs used
Reconstruction of thermal response functions^25^	27 days in March 2024 of all BHEs
Comparison and validation of four BHE modeling approaches^11^	Two one-month periods and a five year period of two BHEs
Investigation on connection pipes capacity in operating BHE fields^15^	Data of all BHEs from July 2018 to June 2019
Verification of a hybrid BHE model^55^	Data of all BHEs from July 2018 to June 2019
Validation of a cloud-based optimized operation strategy^12^	One month of data of all BHEs
Validation of g-function model and parameter estimation for long-term operation optimization^56^	Data from 2015 to 2018

## Methods

### BHE field and monitoring setup

**Fig. 1:**
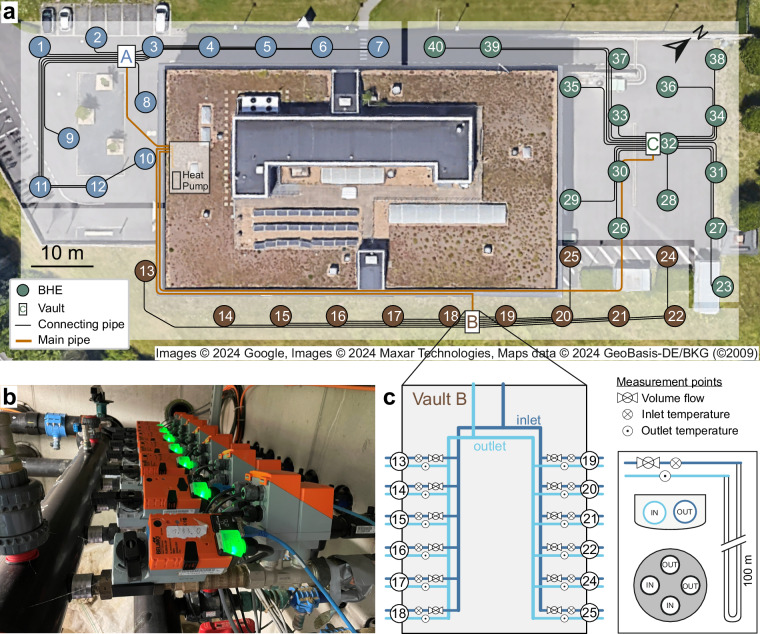
Overview of the BHE field and monitoring system. (**a**) Location of the 40 BHEs (numbered circles) and the three underground vaults (A, B, C), in which the sensors are placed. Horizontal supply and return pipes between the BHEs and the vaults are shown in black, and the main pipes connecting the underground vaults to the building in orange. (**b**) Photography of sensors in underground vault B. (**c**) Schematic overview of underground vault B with sensors at each BHE, as well as cross sections of the horizontal connecting pipes and the BHE.

Monitoring data is recorded at the E.ON Energy Research Center (E.ON ERC), a university building located in Aachen, Germany. The building provides 7800 m^2^ of office space, laboratories, and server rooms on four floors. As a research and demonstration facility, the building is equipped with a variety of energy sources (e.g., combined heat and power plant, condensing boiler) and heating and cooling distribution systems (e.g., radiators, floor heating, concrete core activation)^14^. The core of the building’s energy supply is a heat pump with 80 kW thermal output for heating and 258 kW thermal output for cooling. The heat pump is supplied with low-temperature building waste heat, as well as heat/cold from a BHE field (Fig. 1).

The BHE field consists of 40 double U-tube BHEs with a length of 100 m each. The pipes of the BHEs are made of high-density polyethylene, the backfill material is thermally enhanced grout. The fluid is a water-glycol mixture with 35% ethylene glycol content. The geological environment around the boreholes is homogeneous, with horizontal deposits of clay, silt, and sand. The geometry, material and subsurface properties of the BHEs and their surroundings are given in Table 3.

**Table 3:** Material and geometric properties of the BHE field.

Parameter		Value	Unit
BHE geometry	BHE Length	100	m
	Borehole diameter	0.152	m
	Distance between pipe legs	0.04	m
	Pipe outer diameter	0.032	m
	Pipe wall thickness	0.0029	m
Horizontal pipe geometry (BHE to vault)	Pipe outer diameter	0.04	m
	Pipe wall thickness	0.0037	m
Ground	Average thermal conductivity	2.3	W m^−1^ K^−1^
	Average volumetric heat capacity	2.3 × 10^6^	J m^−3^ K^−1^
Materials	Grout thermal conductivity	2.0	W m^−1^ K^−1^
	Grout volumetric heat capacity	1.0 × 10^6^	J m^−3^ K^−1^
	Pipe thermal conductivity	0.3	W m^−1^ K^−1^
Fluid	Thermal conductivity	0.43	W m^−1^ K^−1^
	Density	1054	kg m^−3^
	Volumetric heat capacity	3800000	J m^−3^ K^−1^
	Dynamic Viscosity	0.0035	Pas

The 40 BHEs are arranged in three sub-fields (Fig. 1) with three underground vaults (A, B, C), to which the respective BHEs are connected via horizontal supply and return pipes. The underground vaults, in turn, are connected to the building’s mechanical room by main supply and return pipes measuring 29 m, 91 m, and 117 m in length. The pipes linking the borehole heat exchangers to the underground vaults range from 2 m (for BHE 32) to 60 m (for BHE 13), resulting in a total length of 900 m for all BHEs, including both supply and return pipes^15^. The horizontal pipes are not insulated and buried in sand-filled trenches, whose depth ranges between 1–1.5 m. In the control room, two hydraulic pumps with 6 kW electrical power operate alternately to generate the required volume flow of up to 1200 l/min in the extensive pipe network.

### Sensors and their uncertainties

Inside the underground vaults, each BHE is equipped with a sensor for the inflow and outflow temperature, as well as the fluid flow rate (Fig. 1). To measure **fluid temperatures**, two-wired Pt1000 resistance thermometers were inserted into downward-facing thermal wells (sockets) at the inlet and outlet pipes. The thermometers correspond to class B of DIN EN 60751^16,17^. Compared to four-wired thermometers, two-wired thermometers are less accurate and strongly dependant on calibration, especially when the cable is long^18^. To account for this, the cables of all sensors have the same length of exactly 1 m^17^.

The uncertainty E of the temperature sensors is commonly described as a limit deviation (i.e., the maximum allowable deviation from the nominal value) by the manufacturers, denoted as a combination of a constant and a temperature dependent part:

1 $${E}_{{T}_{in,out}}=\pm \,\mathrm{(0.3}\,K+\mathrm{0.005|}T|\mathrm{)}.$$

The capital *E* is used to denote absolute uncertainty, while a lowercase *e* refers to relative uncertainty^19^. The limit deviation is commonly reduced by sensor calibration, i.e., aligning the sensor reading with a known standard or reference. The measurement resolution (i.e., the smallest detectable change in temperature that the sensors can reliably discern) is 0.05 K. To calculate performance metrics, the temperature difference $\varDelta T={T}_{out}-{T}_{in}$ is the quantity of interest, not the absolute measured temperature. To measure Δ*T* with a high resolution, the temperature sensors were calibrated in pairs to reduce the sensor discrepancy. A study on the calibration of the sensors conducted in 2018^20^ has shown that, while the absolute measured temperature can be up to 0.126 K off, the relative temperature difference of the sensor pairs has a maximum deviation of 0.05 K. Thus, the pairwise sensor calibration reduced the absolute error of the temperature difference to

2 $${E}_{\varDelta T}=\pm \,0.05\,K,$$

assuming the same temperature dependence for both sensors. The **volume flow** *V* is recorded by Belimo Energy Valves using ultrasonic travel-time flow meters. Corresponding to Class 2 of the European norm EN 1434^21^, the sensors have a relative limit deviation not higher than

3 $${e}_{V}=\pm \,5\,\mathrm{ \% }.$$

### Monitoring system

An elaborate monitoring system with over 8000 data points throughout the building enables real-time data tracking and historical data analysis. The operation of the geothermal field began in November 2011, while data monitoring started in June 2014. Initially, the monitoring platform was SQL-based, taking measurements approximately every 5–20 minutes at irregular intervals. Due to instability and temporary failures^17^, this platform was replaced in May 2018 with a commercial influx-based monitoring platform^20^, which provides higher data resolution and measurement stability. Since then, measurements from the BHEs have been recorded at 30-second intervals. Moreover, the commercial cloud platform allows sending control signals to the Belimo Energy Valves, which enables the testing and implementation of optimized control algorithms^12^.

This article provides reliable and coherent data of a six-year data period, from July 1 2018 to June 30 2024, that was collected by the influx-based monitoring system.

### Raw data and preparation

**Fig. 2:**
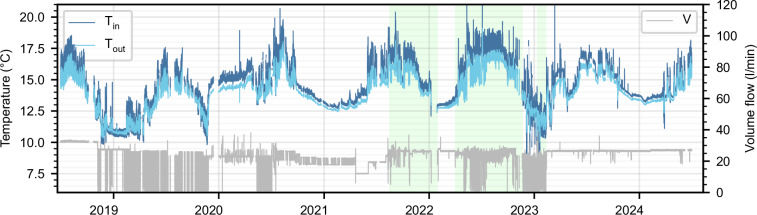
Raw data of BHE 16 for the entire published period. Inlet and outlet temperatures are shown in blue (left axis), the volume flow is shown in gray (right axis). Data periods in which the operation was optimized are highlighted in green.

**Fig. 3:**
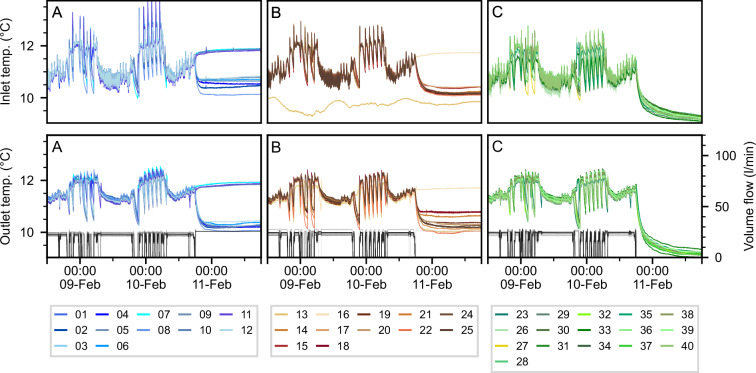
Raw inlet (top row) and outlet (bottom row) temperatures grouped by underground vault of three days in February 2023.

As an example of the raw data, Fig. 2 shows the inlet and outlet temperature of one BHE, as well as its volume flow rate, for the published period. Yearly seasonal fluctuations of the temperature are visible. During approximately 17 months, the operation strategy of the field was optimized using a model predictive controller^12^. Figure 3 shows T_in_, T_out_ and V, measured over three days in February 2023 for all 40 BHEs, grouped by underground vault. The figures show some issues with the raw data: i) data is recorded during no-flow times, and ii) data of some BHEs is disturbed due to sensor malfunctioning, for example BHE 13.

For those reasons, the raw data is processed and resampled to exclude non-representative data periods and decrease the dataset size. The processed dataset is provided alongside the raw data, and processing steps may be repeated with custom settings using the scripts provided in the github repository (https://github.com/elimh/ERC_BHEfield_Data_Code). The steps conducted to obtain the prepared dataset (masking, resampling, outlier identification) are described in the following.

#### Masking of non-representative periods

**Fig. 4:**
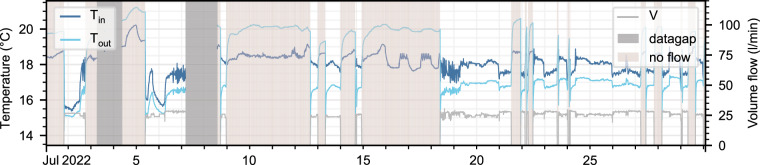
Data of a one month period in July 2022 of BHE 40, with data that will be masked highlighted in gray (masked due to datagap) and red (masked due to no flow period).

To ensure the data accurately represents the thermal processes in the BHEs, periods in which the data is not representative are masked, i.e., overwritten with “no data” (NaN) values. This includes times when the volume flow rate in a BHE is zero, as the temperature reflects the fluid at rest in the pipes, influenced by conductive heat transfer with the surroundings^22^. Once the flow resumes, the fluid temperatures is discarded until the fluid has completed one cycle through the pipe. Assuming a measured throughput of 30 liters per minute, the fluid requires about 9 minutes to flow through the double-U tube BHE, plus an additional 30 seconds to five minutes for the single horizontal pipes ranging from 3 meters (BHEs 18, 19, 32) to 60 meters (BHE 13) to be completely renewed. Thus, the first 10 to 15 minutes after a BHE switches on are masked, depending on the length of the horizontal pipes. The same procedure is applied to periods during and after data gaps in the flow rate, as the usage of the BHE during these times is unknown. An example of a data section with off-times and data gaps, both of which would be overwritten with NaNs, is shown in Fig. 4.

#### Resampling

**Fig. 5:**
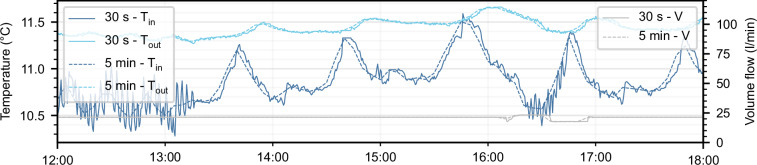
Comparison of the raw data in its initial 30-s time resolution and the resampled 5-min dataset.

As an additional processing step, the data with a 30-second time resolution is resampled to five-minute intervals to reduce the dataset size by 90%. While a 30-second resolution may be necessary for optimal control problems, modeling applications or BHE temperature evolution analyses typically require resolutions of 10–30 minutes^23^. Here, the fluid takes 10–13 minutes to flow through a BHE, making 5-minute intervals a reasonable compromise to capture small-scale temperature changes while effectively reducing the size of the dataset. For resampling, we calculate the mean value of ten timesteps and assign the value to the mean time of the 5-minute period. This resampling method flattens periodic small-scale temperature variations in the data caused by the measurement resolution of 0.05 K, while preserving the general temperature evolution (Fig. 5).

#### Identification of disturbed temperature measurements

**Fig. 6:**
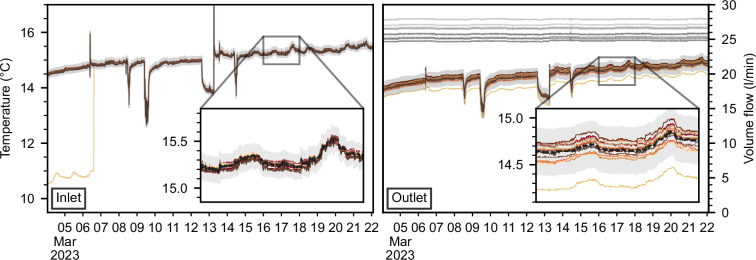
Fluid temperatures and volume flow in vault B, with the windows applied for outlier detection shown in light gray. The dotted black lines are the median of the data. See Fig. 3 for the color legend of the BHEs.

In addition to excluding non-representative measurements, we conducted a plausibility check to identify and exclude sensors that measured disturbed temperatures. Due to oversight mistakes during hardware selection and installation, the PT1000 sensors were too short to be properly secured with the fastening screw inside the thermal sockets. Because the sockets were oriented upside down, some sensors slowly moved out of their valves, causing the measured temperature to be influenced by the air temperature. Sensors that fell out completely were easy to identify and fix (e.g., the inlet temperature of BHE 13 in Fig. 6). It was more difficult to spot sensors that detached slowly. As of February 15, 2024, all temperature sensors are properly attached.

Prior to this date, we identified measurements influenced by this issue by applying the following reasoning. First, all BHEs within one underground vault are fed with the same fluid from the main pipes of the building, so the inlet temperature sensors should record the same inlet fluid temperature, within measurement uncertainty, in each underground vault. In contrast, the outlet temperature may vary due to the heat transfer process in the borehole. If the inlet or outlet temperature measurements of some sensors showed a different trend than the others (e.g., BHE 13 in Fig. 6) or deviated significantly from the inlet/outlet temperature of other sensors in the same vault beyond measurement uncertainty (e.g., BHE 26 in Fig. 6), the measurement can be considered disturbed.

To automatically detect disturbed sensors and outliers in the data, we applied a custom filter to the data. The filter calculates the median of the inlet and outlet temperatures for each underground vault, and then determines the mean absolute error (MAE) of the measurements relative to that median for one-month data periods. If the MAE exceeds a threshold, the sensor is considered disturbed, and the data is masked. We experimented with several threshold combinations and ultimately applied a threshold of 0.12 K for the inlet temperatures and 0.24 K for the outlet temperatures. The higher threshold for the outlet temperatures accounts for the heat transfer process occurring in the BHE and the potential slight variations in volume flow. This procedure is not possible during periods when only a fraction of the BHEs were operated (e.g., from June 1, 2022, to December 31, 2022). During this time of optimized operation, the BHE runtime was significantly reduced from 40 to around 10 BHEs, making it impossible to create a meaningful median. Consequently, periods of optimized operation are excluded from this analysis. However, sensors identified as faulty before and after these periods are assumed to be faulty during this period as well.

The filter reveals that the inlet temperature sensors for BHEs 26, 29, 33, and 35 are disturbed, as well as both the inlet and outlet temperatures of BHE 13. The disturbed sensors and their respective timeframes are summarized in Table 4. BHE 26 exhibits a constant misfit of approximately 0.29 K compared to the other inlet temperatures in vault C over the entire period, as indicated by a small interquartile range (IQR). In contrast, the other BHEs displayed a seasonal trend in their misfit, indicated by a higher interquartile range. This suggests that all faulty sensors, except for BHE 26, partly disconnected from the socket, whereas BHE 26 was likely forgotten during calibration.

**Table 4:** Overview of sensors that have a misfit higher than 0.12 K for inlet temperatures and 0.24 K for outlet temperatures compared to the median of the other sensors in the underground vault.

Sensor	From	To	Time (%)	mean MAE	IQR
Probe_13_T_out	2021-02-01	2024-02-15	15	0.3	0.04
Probe_13_T_in	2019-02-01	2023-03-06	70	1.4	1.65
Probe_26_T_in	2018-07-01	2024-06-30	100	0.29	0.03
Probe_29_T_in	2019-02-01	2024-02-15	51	0.26	0.21
Probe_33_T_in	2019-07-01	2024-02-15	65	0.28	0.18
Probe_35_T_in	2019-08-01	2024-02-15	36	0.19	0.05

## Data Records

Both the raw and processed versions of the six-year long high-resolution monitoring data are available at Zenodo^24^. The repository contains the following entries:

- data_raw_30s.zip: A zipped folder of monthly raw data in 30-second intervals in .csv file format. Each file contains three columns (T_in_, T_out_, V) for each of the 40 BHEs, resulting in 120 columns per csv file, plus one for the timestamp. The units of the temperature measurements are °C, the volume flow is given in l/min.
- data_prepared_5min.zip: A zipped folder of monthly processed data in 5-minute intervals in .csv file format. Each csv file has three columns (T_in_, T_out_, V) for each of the 40 BHEs, resulting in 120 columns per csv file, plus one for the timestamp.
- Supplementary_BHE_data.csv: Tabular data providing the X and Y coordinates and the length of the horizontal connecting pipe for each BHE.

## Technical Validation

### Validation of temperature measurements

As previously noted, the measured inlet temperature of BHEs in a single underground vault should be approximately at the same temperature level, while the outlet temperature can vary to some degree due to the thermal exchange process in the ground and the different horizontal pipe lengths^25^. Moreover, the temperature difference between the inlet and outlet of each BHE should be in the same range and depend on the flow rate, which is one of the most influential operation parameters on the temperature difference of a BHE^26^. This reasoning is applied to validate the temperature measurements. For this, we examine a period where the flow rate is constant for all BHE (March 2023) and compare the inlet temperature, the outlet temperature, and the temperature difference for the BHEs grouped by underground vault.

**Fig. 7:**
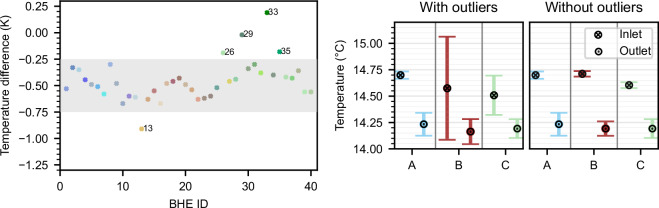
Temperature difference of all BHEs (left) and the mean inlet and outlet temperature (right) of BHEs in one underground with and without disturbed sensors.

Figure 7 (right) shows the mean and standard deviation of the inlet and outlet temperatures by underground vault, including all measurements and excluding the disturbed sensors. With the disturbed sensors included, the mean vault inlet and outlet temperatures show very different standard deviations. Once the faulty sensors are removed, the standard deviation of inlet and outlet temperatures is consistent across the three vaults. Figure 7 (left) illustrates the temperature difference of all sensors for the same data period. Theoretically, all temperature differences within an underground vault should have the same sign and fall within a similar range. While this is the case for most BHEs (indicated by the gray background), the BHEs with faulty sensors are off and even show a different temperature difference sign (BHE 13).

### Validation of volume flow rate measurements

**Fig. 8:**
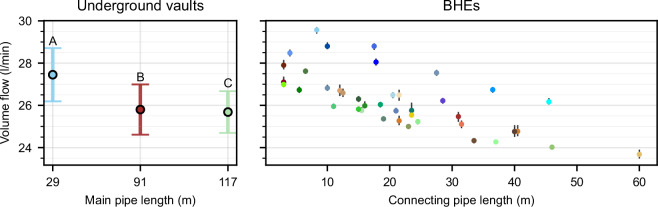
Average volume flow rate for a data period from March 4 to March 22 2023, where the flow rate of all BHEs is constant. The whiskers (left) or gray lines (right) indicate the standard deviation.

To validate the volume flow rate measurements, we examine the volume flow rate as a function of the length of the connecting pipe, as well as the main pipes, for the same data period as before. The flow rate shows a slight variation for each BHE, likely because of the different pipe lengths, leading to increasing pressure loss and hydraulic resistance with increased pipe length. To identify outliers from this picture, Fig. 8 shows the average flow rate as dots and its standard deviation for the three underground vaults (left) and for each BHE (right) as a function of the main pipe length (left) and connecting pipe length (right). The results indicate a trend of decreasing flow rate with increasing pipe length. This trend is consistent, no BHE deviates significantly from this pattern.

## Data Availability

We provide a Github repository (https://github.com/elimh/ERC_BHEfield_Data_Code) containing helper functions to work with the data, scripts to generate the processed data, as well as *Jupyter notebooks*^27^ that were used to create all figures shown in this manuscript except of Fig. 1. This way, the preparation steps are comprehensible and may be repeated with custom parameters. The code is written in *Python 3.10*^28^, package dependencies are part of the core *Python* data science stack (*pandas*, *numpy*, *matplotlib*).
