# Publisher Correction: Six years of high-resolution monitoring data of 40 borehole heat exchangers

**DOI:** 10.1038/s41597-025-04830-2

**Published:** 2025-03-20

**Authors:** Elisa Heim, Phillip Stoffel, Dirk Müller, Norbert Klitzsch

**Affiliations:** 1 RWTH Aachen University, Computational Geoscience, Geothermics, and Reservoir Geophysics, Aachen, 52074 Germany; 2 E.ON Energy Research Centre, RWTH Aachen University, Institute for Energy Efficient Buildings and Indoor Climate, Aachen, 52074 Germany

**Keywords:** Geothermal energy, Environmental sciences, Geothermal energy, Environmental sciences

Correction to: *Scientific Data* 10.1038/s41597-024-04241-9, published online 17 December 2024

In Table 3 of this article, the Ground average volumetric heat capacity and Grout volumetric heat capacity were incorrectly given as ‘2.3 × 10^−6^’ and ‘1.0 × 10^−6^’ but should have been ‘2.3 × 10^6^’ and ‘1.0 × 10^6^’, respectively.

